# Objective Assessment of Retinal Artery Macroaneurysm With Optical Coherence Tomography Angiography

**DOI:** 10.7759/cureus.32328

**Published:** 2022-12-08

**Authors:** Madihah Mohd Lokman, Mae-lynn Catherine Bastion, Jemaima Che Hamzah

**Affiliations:** 1 Department of Ophthalmology, Faculty of Medicine, Universiti Kebangsaan Malaysia, Cheras, MYS; 2 Department of Ophthalmology, Faculty of Medicine, Universiti Kebangsaan Malaysia, Kuala Lumpur, MYS

**Keywords:** objective assessment, angiography, retinal vessels disease, retinal artery macroaneurysm, octa

## Abstract

Retinal artery macroaneurysm (RAM) is an acquired retinal arteriole dilatation with vision-threatening complications. Diagnosis of this condition can be made clinically, supported by multi-modal imaging modalities, commonly optical coherence tomography (OCT) and dye-based angiography studies which show the lesion itself and the complications to the adjacent retina. We report a case of an 83-year-old patient with renal impairment who had the diagnosis and monitoring of RAM done using optical coherence tomography angiography (OCT-A) as an alternative to conventional fluorescein angiography. This case highlighted the use of OCT-A using Cirrus 5000 with AngioPlex (Zeiss, Jena, Germany) as a useful diagnostic and monitoring tool for RAM with its features that enables objective quantification of the disease activity via vessel and perfusion density pre- and post-laser treatment.

## Introduction

Retinal artery macroaneurysm (RAM) is an acquired dilatation of the retinal arterial vasculature, usually involving the first three branches of the arteriolar tree, with the second-order arteriole being the most common [1]. They may occur with varying degrees of haemorrhage, oedema, and exudations. Risk factors include being more than 60 years of age, female gender, arteriosclerotic disease, and systemic hypertension [2]. Hypertension and older age lead to hyaline degeneration of the vascular walls, loss of autoregulatory tone and elastic recoil, and arterial dilatation [3]. RAM may undergo spontaneous involution in up to 75% of cases [4]. However, one-third of patients will develop vascular leakage and retinal oedema which require prompt treatment to prevent permanent central vision loss [5]. 

Different imaging modalities play a significant role in helping to establish the diagnosis of RAM, monitor disease progression, and respond to treatment. Optical coherence tomography (OCT), fundus fluorescence angiography (FFA), and indocyanine green angiography (ICGA) have been widely used in the diagnosis and management of retinal vascular diseases. A more recent imaging advancement involves the use of optical coherence tomography angiography (OCT-A), a non-invasive imaging modality that provides structural and functional (blood flow) information from different layers of the retina and choroid [6].

This case report describes the use of OCT-A imaging, using the commercially available Cirrus 5000 with AngioPlex (Zeiss, Jena, Germany) in the diagnosis and management of a patient who has multiple comorbidities that prevent the use of the classical dye-based angiography studies.

## Case presentation

**Figure 1:**
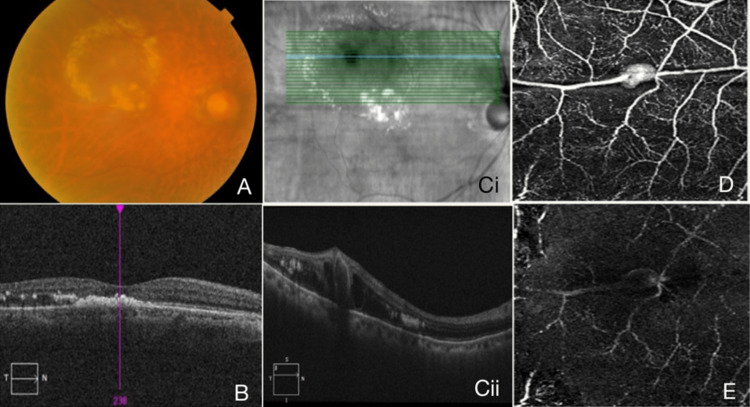
Multimodal Images of the Retinal Artery Macroaneurysm The coloured fundus photography of the right eye at baseline reveals a yellowish oval lesion surrounded by hard exudates that extends into the macula (A). Spectral-domain optical coherence tomography (SD-OCT) across the fovea showed hard exudates with loss of inner segment/outer segment (IS/OS) junction (B). SD-OCT across the lesion showed a cavity with a hyperreflective wall and adjacent retinal thickening with cystic changes (Ci and Cii). The 3x3 mm optical coherence tomography angiography (OCT-A) spanning the RAM is seen in D and E. The OCT-A across superficial capillary plexus segmentation showed high flow within the lesion (D) whilst OCT-A across deep capillary plexus segmentation reveals no obvious high flow (E) which helps identify the lesion as retinal artery macroaneurysm (RAM) and not a polyp.

An 83-year-old gentleman with a history of chronic hypertension, dyslipidemia, ischemic heart disease, stage 4 chronic kidney disease (CKD), and a history of a recent stroke, presented with a progressive right eye with blurry vision for the past two months which was more noticeable in the centre. However, the patient denies metamorphopsia. There were no relieving or aggravating factors to this symptom. He also reported no other associated eye symptoms. His presenting visual acuity (VA) was counting fingers for his right eye and 6/24 for his left eye. There was no relative afferent pupillary defect. The patient had bilateral cataracts with nuclear sclerosis 2+ and posterior subcapsular cataract 1+. Dilated fundus examination of the left eye was unremarkable whilst his right eye revealed an oval lesion along the superotemporal arcade with surrounding hard exudates (Figure 1A). There was an absence of drusens, no orange nodule, and no hypertensive retinopathy changes such as tortuous arterioles with silver or copper wiring, arteriovenous crossing, or cotton wool spots. Spectral-domain optical coherence tomography (SD-OCT) macula of the right eye showed hard exudates at the fovea (Figure 1B). SD-OCT across the lesion showed a round cavity with a hyperreflective wall and central hyporeflective lumen (Figure 1C). OCT-A was performed in view of his renal impairment and was able to show a distinct fusiform lesion in the superficial layer corresponding to the oval lesion seen during fundus examination (Figure 1D). Fundus findings and features on the multimodal imaging performed were consistent with right eye superotemporal exudative RAM.

**Figure 2:**
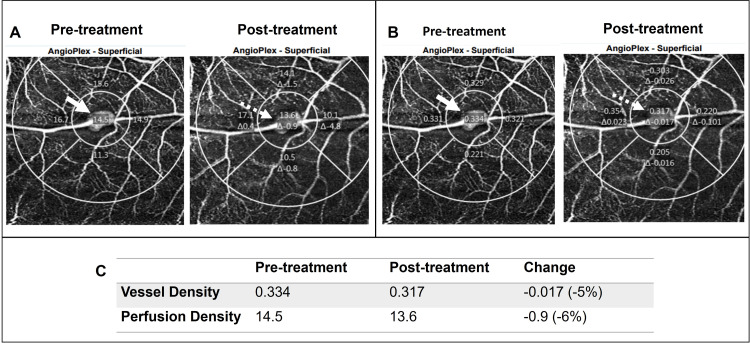
Angiography Changes Pre- and Post-Argon Laser Treatment The perfusion density was measured across the retinal artery macroaneurysm (RAM) pre- (solid white arrow) and post-argon laser treatment (dotted white arrow) (A). Vessel density was measured across the RAM pre- (solid white arrow) and post-argon laser treatment (dotted white arrow) (B). There was a 5% reduction in vessel density and a 6% reduction in perfusion density observed as early as two hours post-laser treatment (C).

The patient was promptly treated with argon green laser photocoagulation performed with an Area Centralis lens; 70 shots x 0.15s x 200 um x 220-280 mW were applied to the RAM and the adjacent area of retinal thickening to produce a light grey burn with the aim to seal-off the macroaneurysm and resolve the surrounding exudates. The argon laser treatment showed an effect on the RAM as early as two hours post-laser treatment as shown by the reduction in vessel and perfusion density, derived from the OCT-A (Figure 2). 

**Figure 3:**
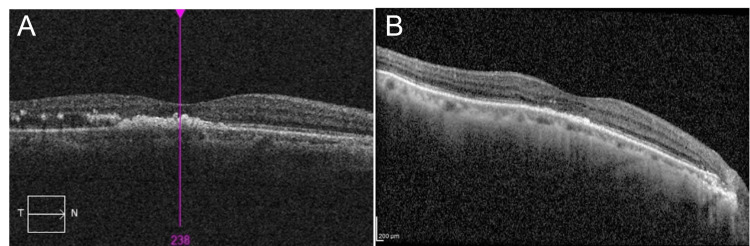
SD-OCT Imaging Showing Macula of the Right Eye Pre- and Post-Argon Laser Treatment SD-OCT imaging showing macula at six weeks post laser photocoagulation displayed a reduced amount of exudates and improved organisation of neurosensory layers at the fovea (B), as compared to baseline at presentation (A). Vision improved to 6/60 SD-OCT: Spectral-domain optical coherence tomography

At six weeks post presentation and treatment, right eye vision improved to 6/60, N48. SD-OCT imaging of the right eye showed reduced subfoveal hard exudates (Figure 3). The patient also underwent cataract extraction and intraocular lens implantation for his left eye. Post-surgery, his left eye vision improved to 6/7.5. To date, our patient is well with no worsening or recurrence of RAM.

## Discussion

OCT and FFA are widely used to support the diagnosis of RAM and to monitor treatment response. Active RAM on OCT can be visualized as a circular or oval lesion in the intraretinal layers with a dark lumen surrounded by a hyperreflective wall [7]. The lumen may be fully hyperreflective, and the size of the lesion is smaller post-treatment. The consequences of RAM to the adjacent retina such as the presence of fluid and hard exudates are clearly visualized in OCT. Swept-source optical coherence tomography (SS-OCT) possesses a feature that enables RAM to be delineated and measured on en-face images [8]. On FFA, RAM can be seen as a fusiform or saccular dilatation. FFA can also show the rate of filling and leakages from the RAM. However, FFA is invasive and requires the use of dye which renders it unsuitable for patients with renal impairment and may also incite allergic reactions.

OCT-A provides a good alternative to dye-based angiography. The method is non-contact, non-invasive, quick to perform and avoids the risks of dye use. OCT-A is superior for its capability of capturing 3D flow data that are compressed to produce 2D en-face images or slabs representing flow in specific retinal and deeper choroidal layers. Pathologies in the retinal and choroidal layers can be visualized without the need for separate tests like in dye-based angiography where FFA is used to study retinal pathologies and ICGA for choroidal pathologies. Retinal artery macroaneurysm has distinct and unique features on OCT-A where the depth of RAM, exact location in relation to the main vessel and level of blood flow can be documented [9], as shown in the present case where the focal fusiform vessel dilatation was shown to arise from the superficial retinal slab, suggestive of RAM instead of arising from a deeper subretinal layer that could be indicative of a polyp or choroidal neovascularization (CNV). As demonstrated in this case, OCT-A allows monitoring of treatment response to laser photocoagulation using perfusion and vessel density profile options [10]. This useful feature of the AngioPlex OCT-A allows disease monitoring and shows treatment responses in RAM as well as many other diseases such as choroidal neovascularization and branching vascular network in which perfusion can indicate a treatment response. However, OCT-A is costly and not readily available in our region. Our centre is one of the few centres that offered the test in Malaysia. OCT-A is also more prone to artifacts and unable to detect low blood flow or leakages.

Treatment with laser ablation has mixed results with some cases having worsening of vision. Hence the treatment is controversial [11]. In the present case, the vision only mildly improved post-laser treatment and this may be explained by the loss of the inner segment/outer segment (IS/OS) junction as seen on OCT images. Earlier presentation and treatment may result in better outcomes. Anti-vascular endothelial growth factor (anti-VEGF) agents may be indicated for hard exudates and oedema in the macula [12]. However, this patient had a recent stroke, so this was not given. The control of the underlying systemic conditions is equally important as it plays a role in determining the treatment options and outcomes. As mentioned, our patient was faced with limited options when he was deemed not to be a suitable candidate for intravitreal anti-VEGF since he had a recent ischemic cerebral vascular accident as a complication to his chronic hypertension and dyslipidemia. Other treatment options include par plana vitrectomy when there is a complication such as vitreous haemorrhage.

## Conclusions

OCT-A is a useful new tool for the diagnosis and monitoring of retinovascular abnormalities, including retinal artery macroaneurysm, without the risks involved in fluorescein angiography. Its capability to quantify angiographic information by providing flow signal, vessel and perfusion densities allows for more objective monitoring of disease progression and treatment response of RAM. OCT with its ability to visualize the intraretinal, and subretinal cystic spaces and hard exudates in exudative RAM complements the OCT-A to objectively monitor the treatment response. For the diagnosis and management of RAM, OCT-A may be at par if not superior to its dye-based angiography counterparts. We believe OCT-A will be the next powerful tool in the management of RAM and eye care practice in general.
